# Cognitive Factor‐Based Selection Increases Power in Alzheimer's Dementia Randomized Clinical Trials

**DOI:** 10.1002/alz70860_099097

**Published:** 2025-12-23

**Authors:** Julia W. Gallini, Zachary H. Baucom, Yorghos Tripodis

**Affiliations:** ^1^ Boston University School of Public Health, Boston, MA, USA

## Abstract

**Background:**

Alzheimer's dementia (AD) is of increasing concern as populations achieve longer lifespans. Many of the recent failed AD clinical trials had a low number of AD events and may have been underpowered. Previous trials have attempted to address this issue by requiring signs of cognitive decline in brain imaging for trial enrollment. However, this method systematically excludes people of color and those without access to healthcare and results in a selected sample that is not representative of the target patient population. We therefore propose the use of a predictive model based on cognitive test scores only to enroll cognitively normal yet high risk participants in a hypothetical clinical trial.

**Methods:**

Cognitive test scores are a widely accessible tool so their use in enrollment would be less likely to exclude marginalized populations than biomarkers, such as imaging, which are overwhelmingly available to exclusively high‐income patients. We developed a novel longitudinal factor model to predict AD conversion within a 3‐year window based on data from the National Alzheimer's Coordinating Center.

**Result:**

Through simulation we demonstrate that our predictive model provides substantial improvements in statistical power and required sample size in hypothetical clinical trials across a range of drug effects when compared to other methods of subject selection.

**Conclusions:**

Predictive modeling using our novel factor model has the potential to improve efficiency in enrollment in AD clinical trials while allowing for a diverse subject set and improved representation of underrepresented groups in AD trials.

